# CD24 associates with EGFR and supports EGF/EGFR signaling via RhoA in gastric cancer cells

**DOI:** 10.1186/s12967-016-0787-y

**Published:** 2016-02-01

**Authors:** Wenjie Deng, Luo Gu, Xiaojie Li, Jianchao Zheng, Yujie Zhang, Biao Duan, Jie Cui, Jing Dong, Jun Du

**Affiliations:** 1 Cancer Center, Nanjing Medical University, 140 Hanzhong Road, Nanjing, 210029 Jiangsu China; 2 Department of Physiology, Nanjing Medical University, Nanjing, 210029 Jiangsu China; 3 Department of Biochemistry and Molecular Biology, Nanjing Medical University, Nanjing, 210029 Jiangsu China; 4 Epidemiology and Biostatistics and Ministry of Education (MOE) Key Laboratory for Modern Toxicology, Nanjing Medical University, Nanjing, 210029 Jiangsu China

**Keywords:** CD24, EGFR, RhoA, Gastric cancer

## Abstract

**Background:**

CD24, a mucin-like membrane glycoprotein, plays a critical role in carcinogenesis, but its role in human gastric cancer and the underlying mechanism remains undefined.

**Methods:**

The contents of CD24 and epidermal growth factor receptor (EGFR) in gastric cancer cells (SGC-7901 and BGC-823) and non-malignant gastric epithelial cells (GES-1) were evaluated by Western blotting assay. Cellular EGFR staining was examined by immunofluorescence assay. Cell migration rate was measured by wound healing assay. The effects of depletion/overexperssion of CD24 on EGFR expression and activation of EGF/EGFR singaling pathways were evaluated by immunofluorescence, qPCR, Western blotting and flow cytometry techniques. RhoA activity was assessed by pulldown assay. CD24 and EGFR expression patterns in human gastric tumor samples were also investigated by immunohistochemistry staining.

**Results:**

CD24 was overexpressed in human gastric cancer cells. Ectopic expression of CD24 in gastric epithelial cells augmented the expression of EGFR, while knockdown of CD24 in gastric cancer cells decreased the level of EGFR and cell migration velocity. To further explore the mechanisms, we investigated the effect of CD24 expression on EGF/EGFR signaling. We noticed that this effect of CD24 on EGFR expression was dependent on promoting EGFR internalization and degradation. Lower ERK and Akt phosphorylations in response to EGF stimulation were observed in CD24-depleted cells. In addition, we noticed that the effect of CD24 on EGFR stability was mediated by RhoA activity in SGC-7901 gastric cancer cells. Analysis of gastric cancer specimens revealed a positive correlation between CD24 and EGFR levels and an association between CD24 expression and worse prognosis.

**Conclusion:**

Thus, these findings suggest for the first time that CD24 regulates EGFR signaling by inhibiting EGFR internalization and degradation in a RhoA-dependent manner in gastric cancer cells.

**Electronic supplementary material:**

The online version of this article (doi:10.1186/s12967-016-0787-y) contains supplementary material, which is available to authorized users.

## Background

Gastric cancer is the second highest cause of cancer-related mortality worldwide [1]. Although early detection was associated with a reduced risk of gastric cancer death rate [2], gastric cancer is often diagnosed at advanced stages with distant metastases and carries a poor prognosis. In an effort to identify genes whose dysregulation could be linked with the invasive capability of gastric cancer cells, investigators have reported multiple markers that could potentially contribute to the highly metastatic traits of gastric cancer [3]. Overexpression of CD24 was observed in gastric cancer tissue and was associated with increased lymph node metastasis and venous invasion of cancer cells, and CD24 was shown to directly affect gastric cancer cell motility and invasive capability [4, 5].

CD24 is a mucin-like membrane glycoprotein and known as a ligand for *P*-selectin, an adhesion receptor expressed on normal endothelial cell and plateslets [6]. CD24 is also identified as a stem cell marker in intestinal and colonic epithelial cells [7, 8]. Similarly, CD24 has also been shown to be expressed in stem cell subpopulations from primary gastric, nasopharyngeal and colon tumors [9–11]. It is well known that negative expression of CD24 has been characterized as one of the biomarkers of breast cancer stem cells, resulting in functional promotion of breast tumor initiation and progression [12]. Interestingly, some reports also showed that CD24 overexpression is associated with progression of breast cancer [13]. More recent evidence has revealed that CD24 is expressed predominantly by differentiated parietal cells in the murine gastric corpus where it modulates gastric responses to *H. felis* and γ-radiation, so it appears not solely as a stem cell marker, but is also involved in regulating the homeostasis of gastric tissues [14]. Several CD24 downstream effectors have been identified recently. For example, CD24 was shown to increase phosphorylation of FAK and paxillin, and enhance integrin-dependent adhesion in breast cancer cells [15]. CD24 can stimulate STAT3 transcriptional activity via Src and subsequently influence its oncogenicity [16, 17]. In colorectal cancer cells, CD24 was also observed to induce promoter activity and expression of the oncomir miR-21 via Src [18]. Although CD24 exerts its biological impacts based on multiple mechanisms, how CD24 contributes to gastric cancer progression remains largely unknown.

Interestingly, an interaction between glycoproteins and growth factor-mediated signaling has been suggested to play a role in regulating tumor cell growth and survival, which revealed that metastatic tumors upregulate the expression of bulky glycoproteins, and suggested that these glycoproteins would influence transmembrane receptor spatial organization and function [19]. In fact, CD24 may affect the function of receptors such as CXCR4 [20] and HER2 [21], which are normally overexpressed in cancer cells. For example, CD24 was shown to support the expression of HER2 and contribute to decrease the sensitivity of HER2-positive breast cancer cells to lapatinib (HER2-targeted therapy) [21]. EGFR, a homodimer of ErbB1, is of particular importance in gastric cancer, as its level of expression is increased during the progression of tumor progression and is correlated with reduced overall survival [22]. In the present study, we investigated the relevance of EGFR expression in CD24 positive gastric cancer. We here show that CD24 positively regulates the expression of EGFR in gastric cancer cells, and that expression of CD24 supports the EGFR-PI3 K/Akt and EGFR-ERK signaling pathway. Interestingly, in SGC-7901 gastric cancer cells, CD24 was shown to maintain the expression of EGFR through a RhoA-dependent manner. The results obtained in this study clearly established a novel relationship between CD24 and EGF/EGFR signaling in the context of migration regulation, which could be essential in promoting aggressiveness of gastric cancer.

## Methods

### Cells and plasmids

Human gastric cancer cell lines SGC-7901, BGC-823, AGS-1 and non-malignant gastric epithelial cells GES-1 were obtained from the Cell Biology Institute of Chinese Academy of Sciences (Shanghai, China). Cells were cultured in Dulbecco’s modified Eagle’s medium (DMEM, high glucose) (Hyclone, Thermo Scientific, Waltham, MA, USA) supplemented with 10 % (v/v) fetal bovine serum (FBS) (Hyclone) and antibiotics (100 U/mL streptomycin and 100 μg/mL penicillin) (Invitrogen, Carlsbad, CA, USA) in a humidified incubator at 37 °C with 5 % CO_2_. Cells were grown on coverslips for fluorescence staining and on plastic dishes for protein extraction. Cells were made quiescent by serum starvation overnight followed by drug treatment.

Full-length CD24 DNA was amplified using the following primer set, sense: 5′-CCC*AAGCTT*ACCATGGGCAGAGCAATGGT-3′, antisence: 5′-CCG*CTCGAG*AGAGTAGAGATGCAGAAGAG-3′. In these primers, *Hind* III and *Xho*I restriction site sequences are italics. The polymerase chain reaction (PCR) products were cloned into the pCMV-C-HA vector (Beyotime, Nantong, China). The plasmid RhoA-V14 was kindly provided by Dr. Stéphane ORY (Institute of Cellular and Integrative Neurosciences, University of Strasbourg, France).

The cells were seeded in 6-well plates, cultured to 60–70 % confluence, and then transiently transfected with those plasmids by using FuGENE HD Transfection Reagent (Promega, Madison, WI, USA) in serum-free OPTI-MEM according to the manufacturer’s instructions.

### siRNA knockdown studies

The sequences of small interfering RNA (siRNA) for CD24 were as follows: #1, 5′-GGAACUUCCAGGUGUUACUTT-3′, #2, 5′-CCCACGCAGAUUUAUUCCATT-3′, and #3, 5′-GCUGGAGUUUCAUGUACAATT-3′. The sequence of siRNA for RhoA was: 5′-GAACUAUGUGGCAGAUAUCUUdTdT-3′ [23] and the sequence of control siRNA was 5′-UUCUCCGAACGUGUCACGUTT-3′ (GenePharmaCo., Shanghai, China). Cells were transfected with control siRNA, RhoA or CD24 siRNA with Lipofectamine 2000 according to the manufacturer’s instruction.

### Migration assay

SGC-7901 cells were seeded in a 96-well plate. Approximately 48 h later, when cells were 95–100 % confluent, wounding was performed by scraping through the cell monolayer with a 10 µL pipette tip. Medium and non-adherent cells were removed, and cells were washed twice with PBS, and fresh medium with or without EGF (R&D Systems, Minneapolis, MN, USA) was added. Cells were permitted to migrate into the area of clearing for 18 h. Wound healing was photographed microscopically (Carl Zeiss Meditec, Jena, Germany).

For Transwell migration assay, SGC-7901 cells in exponential growth were harvested, washed, and suspended in DMEM without FBS. Cells (2 × 10^5^/200 μL) were seeded into polycarbonate membrane inserts (8 μm pore size) in 24-Transwell cell culture dishes. Cells were allowed to attach to the membrane for 30 min. The lower chamber was filled with 600 μL DMEM with EGF. Cells were permitted to migrate for 6 h. After the incubation, stationary cells were removed from the upper surface of the membranes. The cells that had migrated to the lower surface were fixed and stained with 0.1 % crystal violet. The number of stained cells was counted in photos taken by Nikon TS100 (Tokyo, Japan).

### RT-qPCR

Total RNAs were isolated with TRIzol reagent (Invitrogen). Equal amounts of RNA (1 μg) from each sample were used for cDNA synthesis and qPCR was performed on the ABI StepOne™ Real-Time PCR System (Applied Biosystems, Foster City, CA, USA) using GoTaq qPCR Master Mix assay (Promega) and analyzed using StepOne Software v2.1 (Applied Biosystems). 2^−ΔΔCT^ method was used to calculate gene expression levels. For sample loading control, GAPDH was tested. The following primers were used to amplify CD24: 5′-CTCCTACCCACGCAGATTTATTC-3′ (sense) and 5′-AGAGTGAGACCACGAAGAGAC-3′ (antisense); EGFR: 5′-CCCACTCATGCTCTACAACCC-3′ (sense) and 5′-TCGCACTTC TTACACTTGCGG-3′ (antisense); GAPDH: 5′-CATCAGCAATGCCTCCTGCAC-3′ (sense) and 5′-TGAGTCCTTCCACGATACCAAAGTT-3′ (antisense).

### Western blotting and coimmunoprecipitation analysis

Sample protein extraction and concentration determination of whole cells were performed as previously described [24]. Briefly, equal amounts of protein were run on SDS polyacrylamide gels and transferred to nitrocellulose membrane. The resulting blots were blocked with 5 % non-fat dry milk and probed with antibodie against the following proteins: GAPDH (KangChen Bio-tech, Shanghai, China), CD24 (BD Transduction Laboratories, Franklin Lakes, NJ, USA), EGFR and E-cadherin (Santa Cruz Biotechnology, Santa Cruz, CA, USA), RhoA, ERK, P-ERK, Akt, P-Akt (Cell Signaling Technology, Danvers, MA, USA). Protein bands were detected by incubating with HRP-conjugated antibodies (Santa Cruz Biotechnology) and visualized with ECL reagent (Millipore, Billerica, MA, USA). Erlotinib (APExBio, Houston, USA), a selective EGFR inhibitor, was used to block EGFR-mediated signaling.

Coimmunoprecipitation analysis was performed as previously described [25]. Briefly, proteins of indicated cells were pulled down with anti-EGFR antibody at 4 °C for 4 h, and then agarose beads conjugated to protein A+G were added to the supernatant of each sample and incubated with shaking at 4 °C for 1 h. After wash with rinsing buffer six times, agarose-associated protein complexes were eluted using SDS loading buffer and analyzed by Western blotting assays.

### Pulldown assays

For detection of active RhoA, equal amounts of total cellular protein were incubated with GST-RBD (a gift from Dr. Keith Burridge, Department of Cell and Developmental Biology, University of North Carolina, Chapel Hill, NC) beads captured on MagneGST Glutathione Particles (Promega) at 4 °C with constant rotation for 30 min. The beads were washed three times with washing buffer (4.2 mmol/L Na_2_HPO_4_, 2 mmol/L KH_2_PO_4_, 140 mmol/L NaCl, and 10 mmol/L KCl, pH 7.2). At the end of this period, beads were captured by the magnet in a magnetic stand (Promega). After wash three times with ice-cold buffer, beads were resuspended in 2×SDS sample buffer and subjected to Western blotting assays by using anti-RhoA antibody.

### Immunofluorescence assays

Cells used for immunostaining were fixed in ice-cold methanol for 10 min, permeabilized in 0.1 % Triton X-100 and blocked in PBS containing 1 % BSA for 1 h at room temperature. The cells were incubated with antibody against EGFR or E-cadherin at 4 °C overnight followed by incubation with rhodamine-conjugated or FITC-conjugated secondary antibody for 1 h at room temperature within a moist chamber. F-actin was stained with FITC-labeled phalloidin (5 μg/mL) (Sigma) for 45 min at 37 °C. After wash with PBS, the samples were mounted with DAPI Fluoromount G (Southern Biotech, Birmingham, AL). Images were acquired using an Olympus BX51 microscope coupled with an Olympus DP70 digital camera.

### Flow cytometry

A total of 1×10^6^ cells were resuspended in 0.5 mL PBS containing 1 % BSA, and then incubated on ice for 30 min. EGFR antibody (BD Biosciences) was added to cell suspension and incubated on ice for 30 min. Cells were washed and resuspended in 0.5 mL PBS containing fluro-labelled secondary antibody before analysis using a FACS Calibur Flow Cytometer (BD Biosciences).

### Immunohistochemistry

Tumor specimens used were obtained by the First Affiliated Hospital of Nanjing Medical University, the Affiliated Drum Tower Hospital of Nanjing University Medical School, the Affiliated Zhongda Hospital of Southeast University and the Second Affiliated Hospital of Nanjing Medical University (Nanjing, China). Forty-one primary human gastric tumor samples were used for immunohistological staining in our CD24/EGFR expression correlation study. The paraffin sections were deparaffinized and rehydrated. Peroxidase blocking was done with 3 % H_2_O_2_ in methanol for 15 min at 37 °C. Antigen retrieval was performed by transferring the sections into EDTA buffer (pH 8.0). The sections were blocked in goat serum for 1 h and applied with EGFR or CD24 antibody at 4 °C overnight. Then the sections were treated with the secondary antibody for 1 h at 37 °C, and washed in PBS. DAB substrate solution was applied to reveal the color of antibody staining. After counterstaining with haematoxylin, the slides were mounted and the graphs were obtained using an Olympus BX51 microscope. Reagents for immunohistochemistry were all obtained from ZSGB-BIO (Beijing, China). EGFR and CD24 immunostaining was analyzed by evaluation of the percentage of tumor-stained cells and staining intensity, allowing assessment of an immunoreactive score (IRS). The IRS was calculated as intensity of the staining reaction multiplied by the percentage of positive cells as previously described [26, 27].

### Statistical analysis

Statistical analysis was performed using the SPSS statistical software program (Version 19.0; SPSS, Chicago, IL, USA). Data were analyzed by Student’s *t* test. *P* < 0.05 was considered to be significant (two tailed). In immunohistochemistry, the difference in expression level of EGFR and CD24 was according to the IRS score, Pearson correlation test was used to examine association between EGFR and CD24 protein expressions.

## Results

### CD24 is overexpressed and regulates EGFR expression in gastric cancer cells

**Fig. 1:**
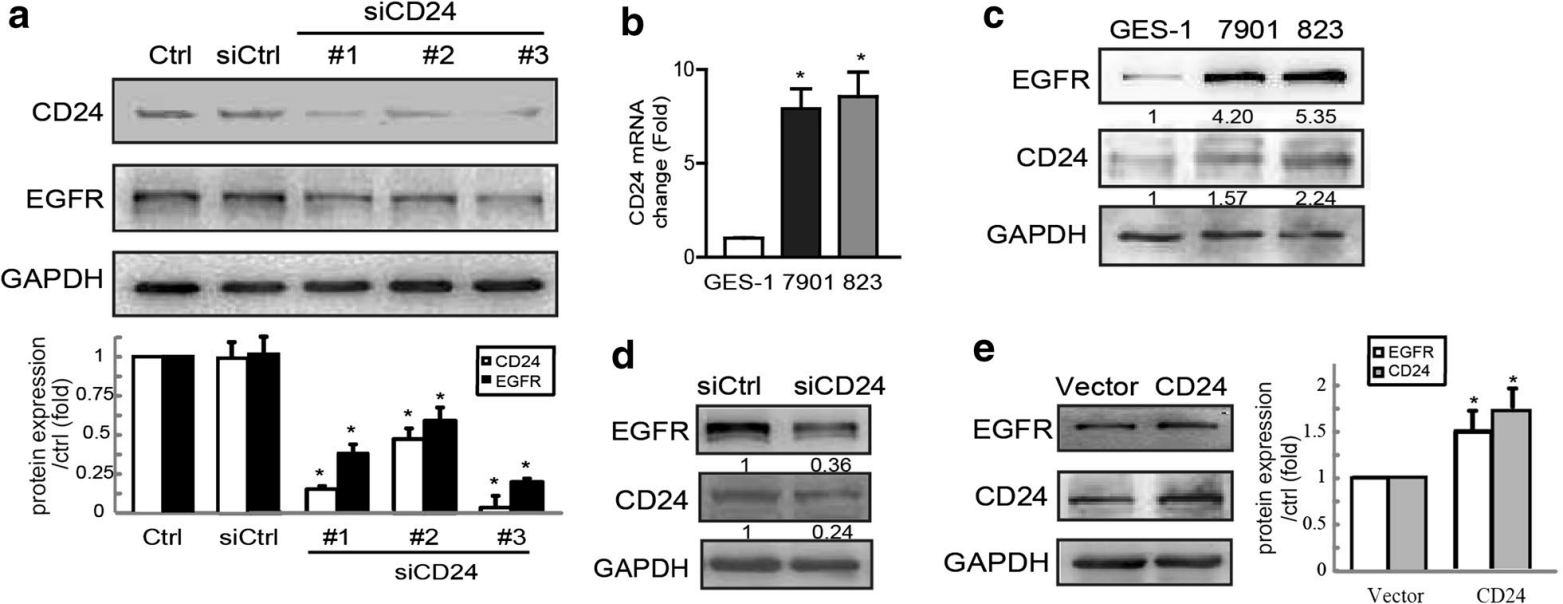
CD24 is associated with EGFR expression in gastric cancer cells. **a** SGC-7901 cells were transfected with negative control siRNA or siRNA specifically targeting CD24 (siCD24). Forty-eight h later, total protein extracts from cells were analyzed by western blotting analysis for CD24 and EGFR expression. Western blot bands corresponding to EGFR and CD24 were quantified and normalized against GAPDH. *Asterisk* P < 0.05 in the siCD24 cells relative to siRNA control cells. **b** qPCR and **c** Western blotting assays of CD24 mRNA and protein expressions in GES-1, SGC-7901 and BGC-823 cells. Data in (**b**) are presented as mean ± SD of 3 determinations *asterisk* P < 0.05 in the cultures of BGC-823, SGC-7901 relative to the cultures of GES-1. **d** BGC-823 cells transfected with control siRNA or siCD24 were lysed, EGFR and CD24 level was determined by Western blotting assays. **e** GES-1 cells were transfected with empty vector or CD24 plasmids, and the total cellular proteins were extracted and analyzed for expressions of EGFR by western blotting assays

To assess the effect of CD24 on total EGFR level, we transfected human gastric cancer cells SGC-7901 with control siRNA or siRNA for CD24. The cells were lysed and total EGFR level was determined by Western blotting. As shown in Fig. 1a, SGC-7901 cells transfected with siRNA against CD24 had a significantly lower level of EGFR than control cells. Two CD24 siRNAs (#1, #3) independently knocked down CD24 expression by more than 80 %, and siCD24 #3 was used in the subsequent experiments. We then evaluated EGFR and CD24 expression in malignant and non-malignant human gastric epithelial cells by qPCR and Western blotting, and found that both EGFR and CD24 expressions at both the mRNA and protein level were increased in gastric cancer cells (SGC-7901 and BGC-823) compared to non-malignant gastric epithelial cells (GES-1) (Fig. 1b, c). To confirm the role of CD24 in regulating EGFR expression, we also performed the same transfection in BGC-823, and found that siCD24 also inhibited the expression of EGFR in those cells (Fig. 1d). In contrast, an increase in the level of EGFR was observed in GES-1 cells (Fig. 1e) and BGC-823 cells (Additional file 1: Figure S1) overexpressing CD24 by Western blotting analyses. These results indicate that CD24 plays a positive role in regulating EGFR expression.

We further analyzed whether CD24 and EGFR were physically associated using co-immunoprecipitation assays, which showed that these two proteins were co-precipitated in both SGC-7901 (Additional file 2: Figure S2A) and BGC-823 cells (Additional file 2: Figure S2B). We therefore next investigated whether there was a physical interaction between CD24 and EGFR. Notably, CD24 and EGFR showed partial co-localization on the surface of BGC-823 cells (Additional file 2: Figure S2C). These data suggested that CD24 was physically associated with EGFR and thereby maintained its localization on the membrane.

### CD24 knockdown affects EGFR function in gastric cancer cells

**Fig. 2:**
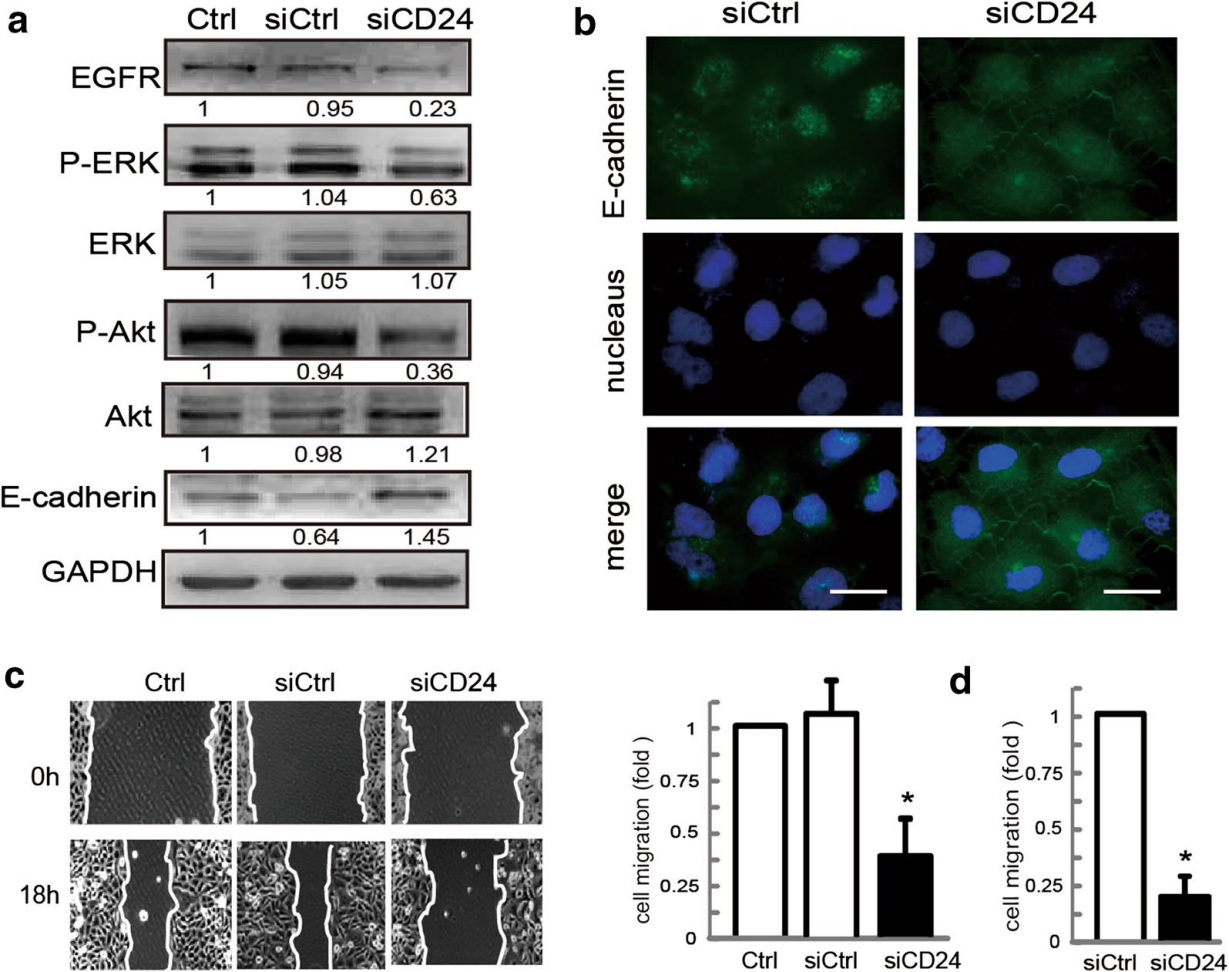
Effects of siCD24 on EGFR downstream proteins and cell migration. **a** SGC-7901 cells transfected with control siRNA or siCD24, and protein levels of EGFR, P-ERK, ERK, P-Akt, Akt and E-cadherin were examined. **b** Representative immunofluorescence images of SGC-7901 cells transfected with control siRNA or siCD24 stained for E-cadherin. *Scale bar*, 10 μm. **c** A representative of wound healing assays in SGC-7901 cells transfected with control siRNA or siCD24 is presented, and the quantification of cell migration rate was performed. Data are presented as mean ± SD of 8 determinations *asterisk* P < 0.05 in the siCD24 cells relative to siRNA control cells. **d** Control and siCD24 SGC-7901 Cell migration rate was also evaluated by Transwell migration assay. Data are presented as mean ± SD of 3 determinations *asterisk* P < 0.05 in the siCD24 cells relative to control cells

The MEK/ERK and the PI3 K/Akt pathways are two of the better-understood EGFR signal transduction pathways involved in cell migration. Since the knockdown of CD24 by siRNA inhibited EGFR expression, we further analyzed the effect of CD24 on EGFR related signaling pathway in gastric cancer cells. Normally, phosphorylation of ERK and PI3 K/Akt keeps high level in SGC-7901 cells. As expected, EGFR downstream effectors ERK and Akt were phosphorylated less in siCD24 transfected cells compared with controls (Fig. 2a). It is well known that erlotinib is a potent selective blocker of EGFR-mediated signaling. Additional file 3: Figure S3 shows that the protein levels of P-Akt and P-ERK markedly increased in CD24-overexpressing GES-1 cells, which was attenuated by erlotinib. Moreover, an increase of P-Akt and P-ERK by EGF stimulation was markedly inhibited by erlotinib. SiEGFR has been reported to reverse EGF-induced E-cadherin low-expression as well as down-regulating subsequent cell invasion in human oviductal epithelial cells [28], so we next analyzed whether knockdown of CD24 affected the total level and distribution of E-cadherin in SGC-7901 cells. Interestingly, our results revealed that the expression of E-cadherin in control cells was weak and large amounts of E-cadherin were localized in the cytoplasm. However, after transfection with siCD24, E-cadherin abundance was obviously increased, and substantial E-cadherin localization was observed, especially in the cellular membrane (Fig. 2a, b). We also found that siCD24-expressing cells exhibited decreased migratory potential than the control cells (Fig. 2c, d). Taken together, these observations suggest that CD24 knockdown impairs EGFR function in gastric cancer cells.

### CD24 affects EGFR internalization and degradation in gastric cancer cells

**Fig. 3:**
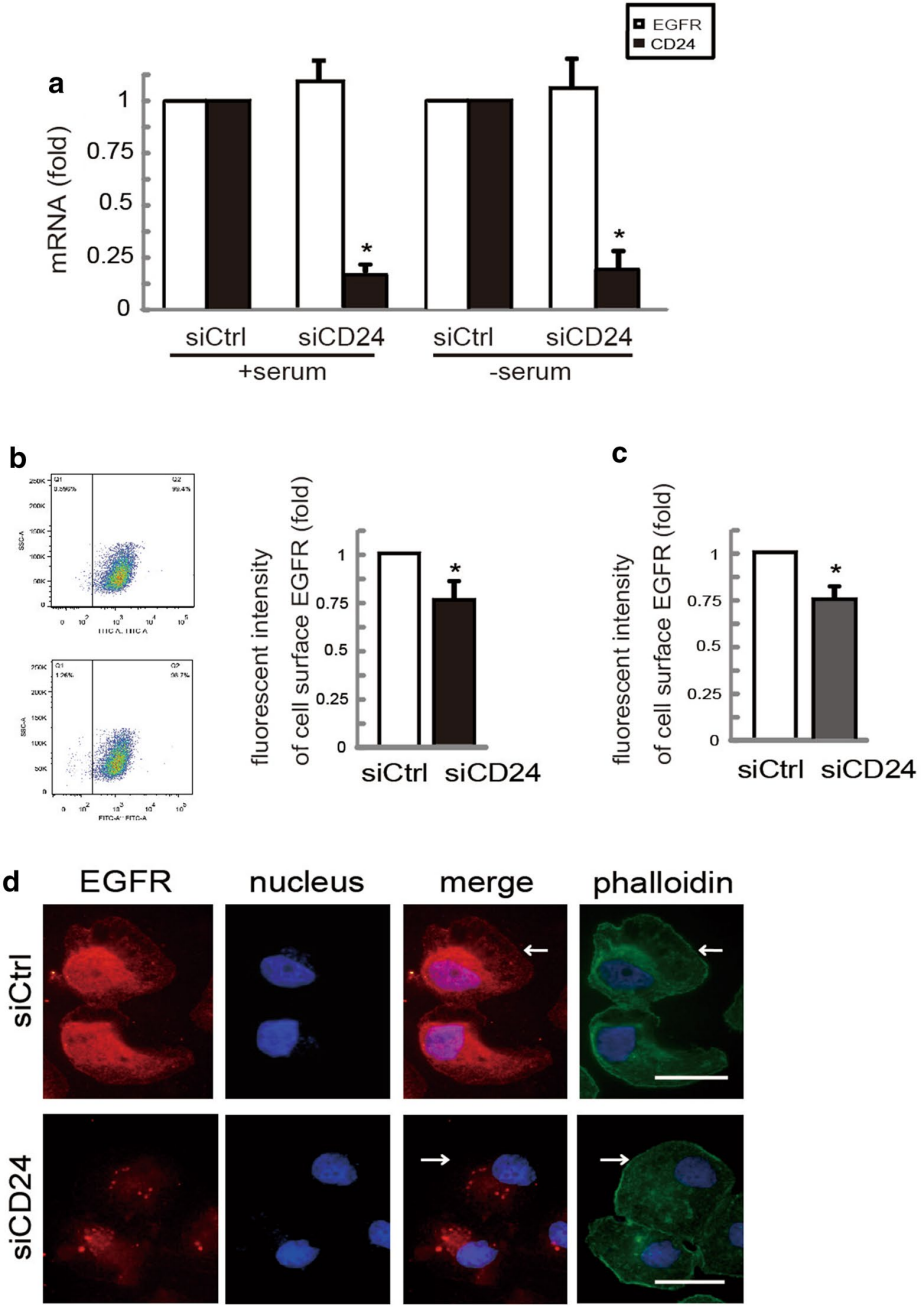
Downregulation of CD24 alters the localization and degradation of EGFR. **a** The mRNA levels of CD24 and EGFR were detected by qPCR in SGC-7901 cells transfected with control siRNA or siCD24. **b** and **c** The level of EGFR on cellular membrane of SGC-7901 cells (**b**) and BGC-823 cells (**c**) was measured using flow cytometry. **d** Representative immunofluorescence images of SGC-7901 cells transfected with control siRNA or siCD24 staining for EGFR and phalliodin. *Scale bar* 10 μM. *Asterisk* P < 0.05 in the siCD24 cells relative to siRNA control cells

To directly test the functional role of CD24 in EGFR expression, we firstly performed quantitative PCR (qPCR) to evaluate the effect of siCD24 on EGFR transcriptional expression. Whereas cells transfected with siCD24 underwent marked reduction in CD24 mRNA expression, the abundance of EGFR mRNA was not affected in serum or serum-free conditions in SGC-7901 cells (Fig. 3a). We thus reasoned that CD24 may modulate EGFR expression by suppressing its distribution in the cytoplasm and the subsequent degradation process. To investigate this possibility, flow cytometry was performed and the results revealed a significant decrease in the staining intensity of EGFR on the membrane in cells transfected with CD24 siRNA in both SGC-7901 (Fig. 3b) and BGC-823 cells (Fig. 3c). In contrast, immunofluorescence staining showed an increase in the staining of EGFR in the cytoplasm and a decrease in EGFR at the membrane in siCD24 transfected cells. FITC-Phalloidin was used to outline cell membrane (Fig. 3d). Collectively, these data suggest that CD24 modulates EGFR subcellular location in gastric cancer cells.

### Knockdown of CD24 downregulates EGFR expression through RhoA in SGC-7901 cells

**Fig. 4:**
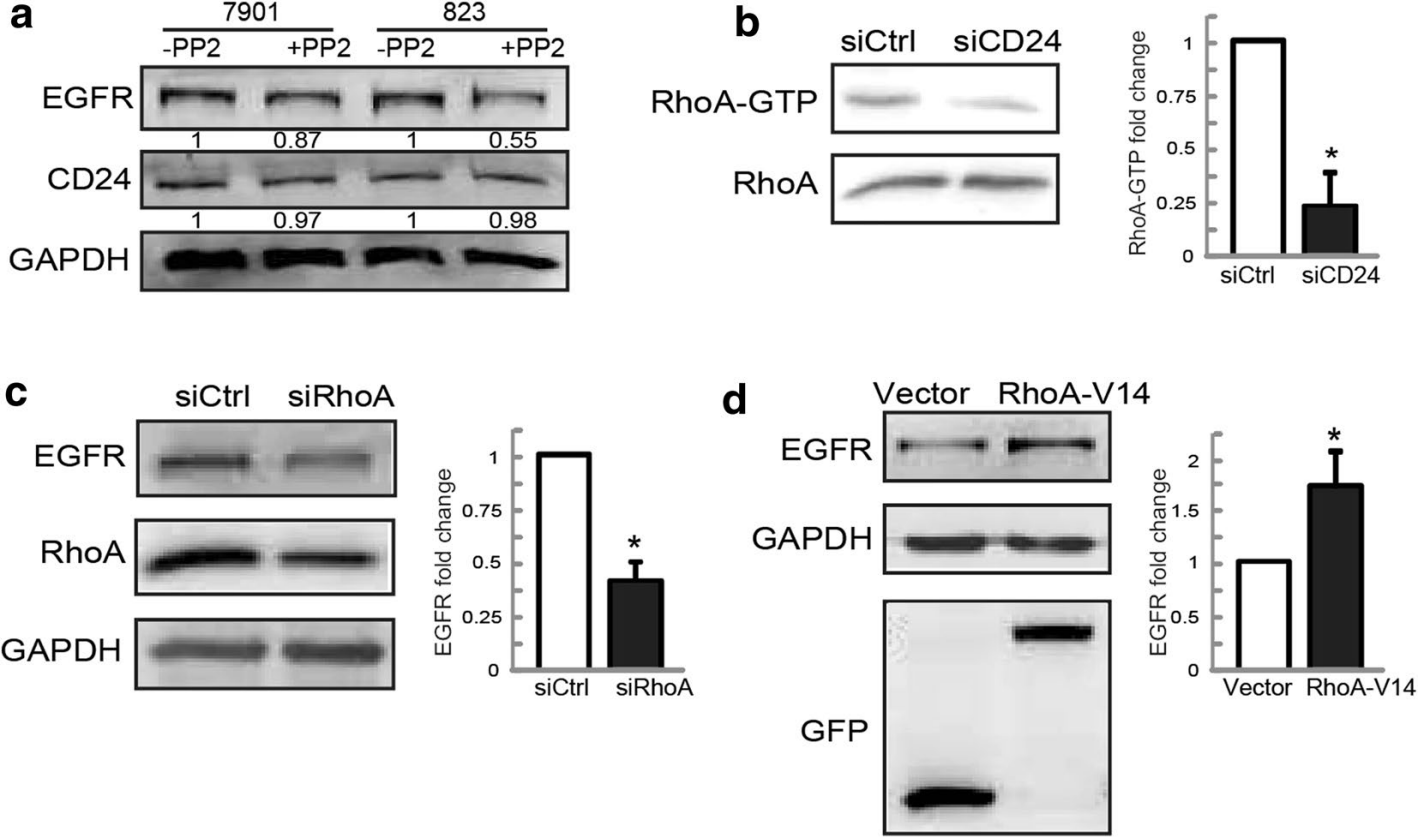
RhoA, but not Src, medicates EGFR expression by CD24 in SGC-7901 cells. **a** SGC-7901 and BGC-823 cells were treated with 20 μM PP2. 30 min later, and total protein extracts from cells were analyzed by Western blotting assays for CD24 and EGFR expression. **b** SGC-7901 cells were transfected with control siRNA or siCD24, and the activity of RhoA was measured. RhoA-GTP bands were quantified and normalized against RhoA level (*graph* on the right). *Asterisk* P < 0.05 in the siCD24 cells relative to siRNA control cells. **c** SGC-7901 cells were transfected with control siRNA or siRhoA, and the expressions of RhoA and EGFR were measured. EGFR bands were quantified and normalized against GAPDH (*graph* on the right). *Asterisk* P < 0.05 in the siCD24 cells relative to siRNA control cells. **d** SGC-7901 cells were transfected with GFP-tagged empty vector or RhoA-V14 plasmids, and the total cellular proteins were extracted and analyzed for expression of EGFR by Western blotting assays. *Asterisk* P < 0.05 in the CD24 overexpressing cells relative to control cells

Although CD24 was proved to be involved in maintaining EGFR expression, the precise mechanism underlying this regulation was poorly known. Considering the importance of Src in regulating receptor internalization [29], and CD24 has been reported to interact with Src [15], in initial experiments, we treated SGC-7901 and BGC-823 cells with PP2 (Alexis Biochemicals), an Src inhibitor, and examined whether PP2 inhibited EGFR expression and CD24 association of EGFR in our experimental system. Although we observed that PP2 did not substantially change the expression levels of CD24 in BGC-823 cells and SGC-7901 cells, significant decrease in EGFR expression was observed in BGC-823 cells, but not SGC-7901 cells (Fig. 4a). We also noticed that, in BGC-823 cell line, the association between CD24 and EGFR was significantly decreased in PP2-treated cells than that in untreated cells. However, PP2 treatment had no effect on the association between CD24 and EGFR in SGC-7901 cells (data not shown), suggesting that Src kinase activity may be required for CD24 to keep EGFR expression in BGC-823 cells, but not in SGC-7901 cells.

The activity of RhoA is known to play an important role in the internalization of EGFR [30]. We thus reasoned that RhoA may be involved in the regulation of EGFR stability downstream of CD24 in SGC-7901 cells. To investigate this possibility, we performed pulldown assay to detect RhoA activity in SGC-7901 cells transfected with siCD24. Interestingly, RhoA activity was decreased after knockdown of CD24, as shown by Western bloting assays (Fig. 4b), while RhoA knockdown by siRNA led to the noticeable decrease of EGFR expression in SGC-7901 cells (Fig. 4c). The protein level of EGFR was significantly higher in RhoA-V14 transfected cells, compared to the controls (Fig. 4d). Taken together, these findings demonstrate that RhoA function is necessary for CD24-mediated EGFR stability in gastric cancer SGC-7901 cells.

### Effects of EGF stimulation on EGFR expression and distribution in gastric cancer cells expressing low levels of CD24

**Fig. 5:**
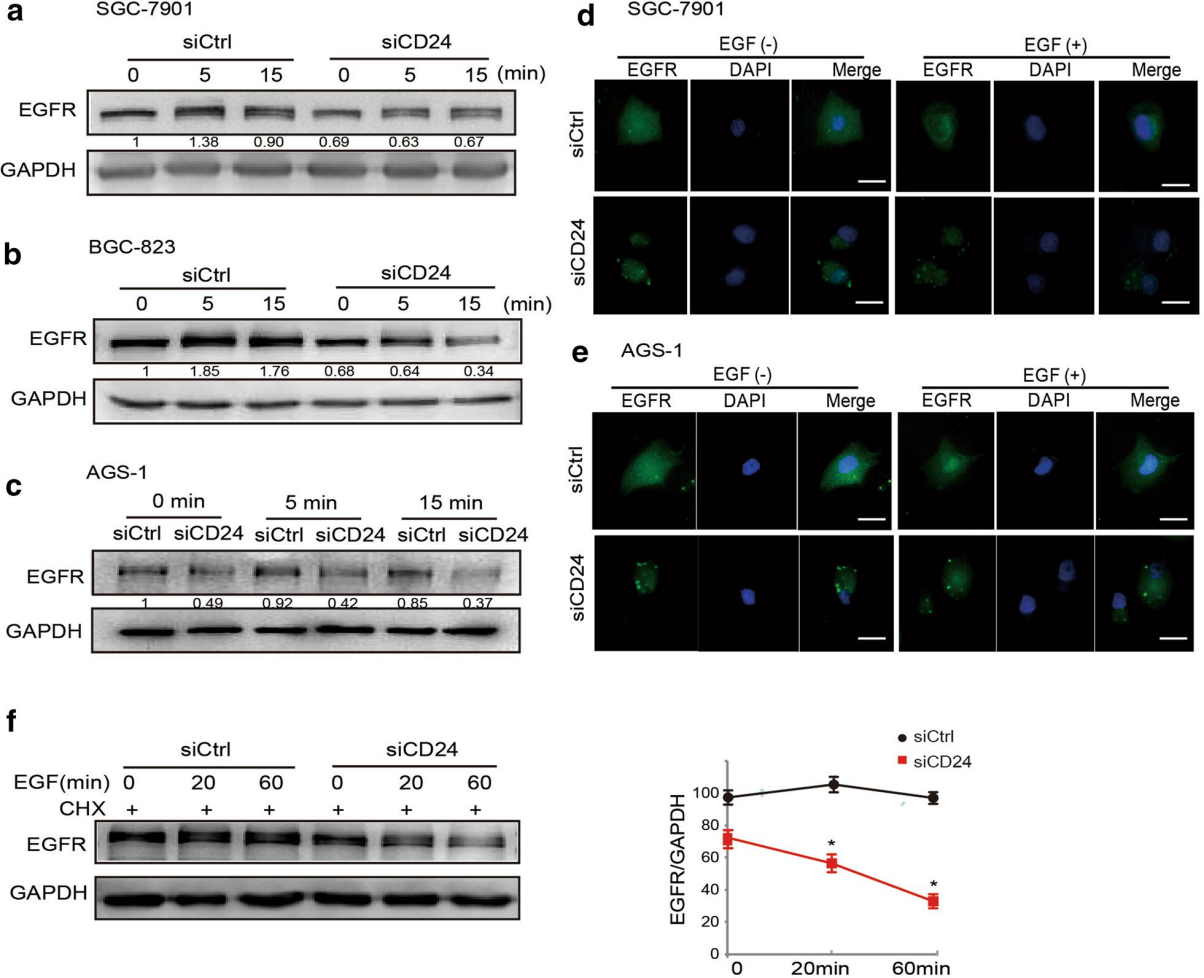
Effects of EGF on EGFR expression and distribution in gastric cancer cells with knockdown of CD24. **a** SGC-7901 **b** BGC-823 and **c** AGS-1 cells transfected with control siRNA or siCD24 were in serum-free media overnight and incubated with EGF 20 ng/mL for indicated times, and then proteins extracted from the lysates were subjected to Western blotting assays to detect the expression of EGFR. GAPDH is used for control. **d** SGC-7901 and **e** AGS-1 cells transfected with control siRNA or siCD24 were in serum-free media overnight and incubated with EGF 20 ng/mL for 15 min, and representative microscopy images of SGC-7901 and AGS-1 cells stained for EGFR are shown. Cell nuclei were labeled with DAPI. *Scale bar* 10 μm. **f** SGC-7901 cells transfected with control siRNA or siCD24 were in serum-free media overnight. In addition to its protein synthesis blocked with cycloheximide (CHX, 10 μg/mL), the cells were then stimulated with EGF (20 ng/mL) for the indicated times. Then, the cells were lysed and EGFR level was determined by Western blotting assays. EGFR bands were quantified and normalized against GAPDH (*graph* on the right). *Asterisk* P < 0.05 in the siCD24 cells relative to siRNA control cells

To further analyze the effect of CD24 on EGFR stability and following signaling pathway, SGC-7901 cells were transfected with siCD24 and stimulated with EGF for different spans of time. As shown in Fig. 5a–c, knockdown of CD24 expression accelerates EGFR degradation in SGC-7901, AGS-1 and BGC-823 cells. Interestingly, ERK and Akt (EGFR main downstream effectors) were also phosphorylated less in siCD24 transfected cells compared with controls (Additional file 4: Figure S4). This observation suggests a decrease in EGFR signaling activation induced by CD24 low-expression. Moreover, depletion of CD24 induced a marked increase in EGFR concentration in the cytoplasm in spite of EGF stimulation in SGC-7901 (Fig. 5d) and AGS-1 cells (Fig. 5e). To discriminate between protein synthesis and degradation, we blocked translation by treating the cells with cycloheximide (CHX). As shown in Fig. 5f, silencing CD24 expression significantly accelerated EGFR degradation stimulated by EGF.

### Expression of EGFR in gastric cancer correlates with CD24 expression

**Fig. 6:**
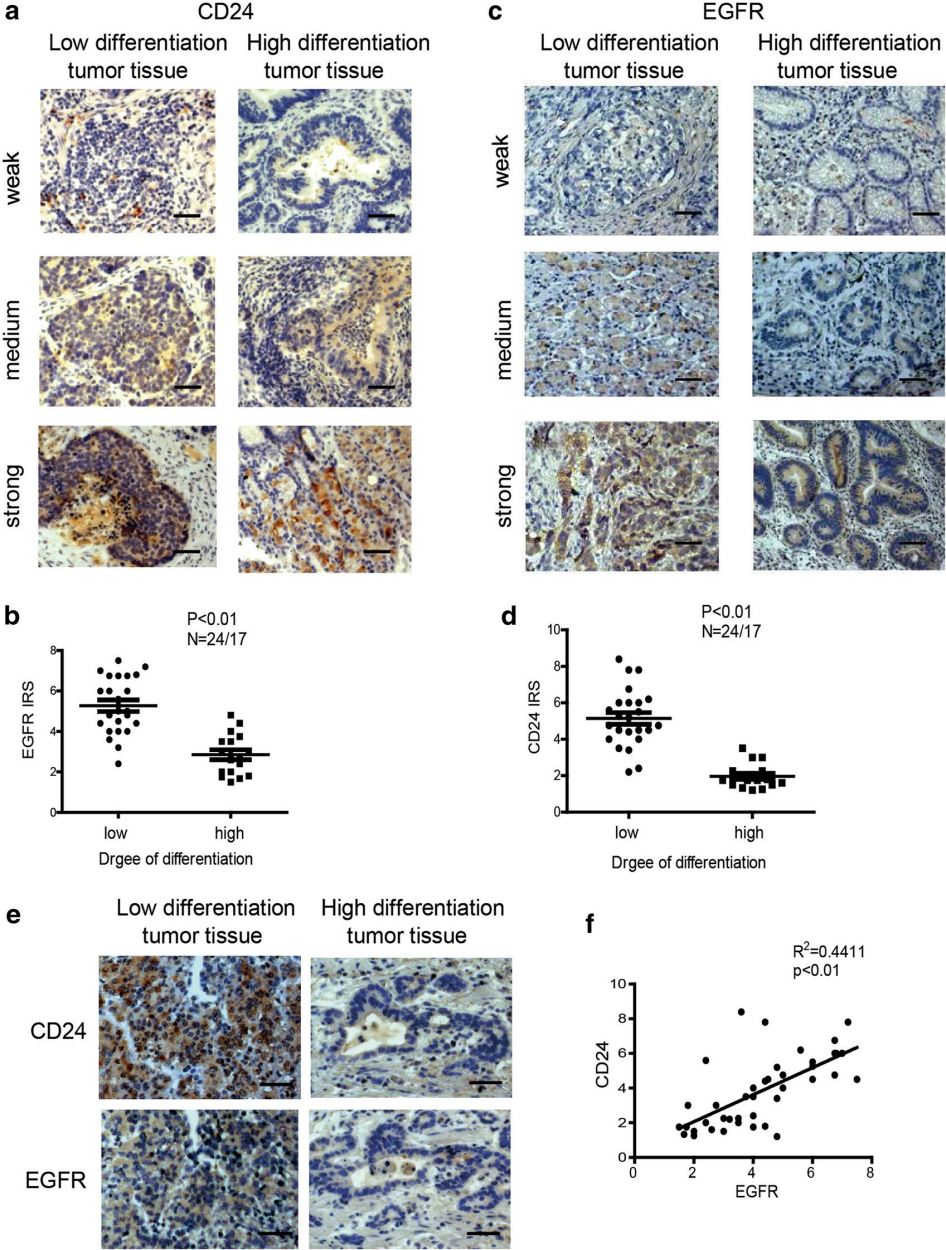
CD24 expression is relative with the low degree differentiation of malignant gastric cancer and has a positive correlation with EGFR. The IRS is calculated as intensity of the staining reaction multiplied by the percentage of positive cells. Based on the IRS values, CD24 and EGFR were scored as weak, medium and strong in the following parts. **a** High and low degree differentiated malignant gastric cancer tissue sections were stained against CD24. *Brown*, CD24; *Blue*, haematoxylin. **b** IRS scores of CD24 according to tumor histological grade. **c** High and low degree differentiated malignant gastric cancer tissue sections were stained against EGFR. *Brown*, EGFR; *Blue*, haematoxylin. **d** IRS scores of EGFR according to tumor histological grade. **e** Using serial sections of the same sample, representative high and low degree differentiated malignant gastric cancer tissue stained for CD24 and EGFR are shown. **f** The *scatterplot* of correlated protein levels between CD24 and EGFR in high and low degree differentiated malignant gastric cancer tissue (n = 41). *Scale bar* 100 μm

To investigate whether our experimental findings could be relevant to the pathogenesis of gastric cancer in humans, we examined CD24 and EGFR expression patterns in high and low degree differentiated gastric cancers. Representative results of CD24 and EGFR immunostaining of gastric cancer are shown in Fig. 6a, c. Among 41 gastric cancer cases, we found that both CD24 and EGFR expression level was markedly increased in low-differentiated tumor tissues when compared with their matched highly-differentiated tumor tissues (Fig. 6b, d). Furthermore, immunostaining of CD24 and EGFR in the samples revealed a positive correlation in expression (r = 0.4411, P < 0.01) (Fig. 6e, f). Overall, the clinical data support our in vitro results that CD24 may play a role as an EGFR supporter to promote gastric cancer progression.

## Discussion

Aberrant activation CD24 signaling contributes to malignant progression of gastric cancer has been demonstrated recently [4, 17]. Here, we investigated the relationship between CD24 and EGFR in gastric cancer cells. We demonstrated here that knockdown of CD24 accelerates internalization and decreases membranous stability of EGFR, leading to an impaired EGFR signaling cascade as well as cell migration. Moreover, we found that CD24-mediated activation of RhoA is required for maintaining EGFR expression. Moreover, analysis of gastric cancer specimens also showed a positive correlation between CD24 and EGFR expression. Together, our results indicate that CD24 plays an important role in regulating EGFR and EGFR-initiated signaling in gastric cancer.

EGFR is a well-studied transmembrane receptor that activates intracellular signaling cascades and controls most vital cellular processes such as survival and cell motility [31]. Accordingly, upregulation of EGFR expression is central to gastric cancer cell metastatic behavior [32]. In the present study, the fact that depletion of CD24 leading to low expression of EGFR in gastric cancer cells attracted our attention. Because the gastric cancer tissue samples we studied here were from China, we used the two gastric cancer cell lines derived from Chinese gastric carcinoma patients to study the regulation of EGFR by CD24. Consistent with the in vivo data, which showed a positive correlation between CD24 and EGFR levels in gastric cancer specimens, our in vitro results revealed that CD24 expression was higher in SGC-7901 and BGC-823 cells, which also showed remarkably increased expression of EGFR than normal gastric epithelial cells. Moreover, we also noticed that overexpression of CD24 elevated EGFR expression in GES-1 gastric epithelial cells. Those results revealed a further mechanism worth exploring for the high amount of EGFR in gastric cancer, especially as CD24 expression itself correlates with a poor prognosis [33, 34].

Although CD24 was detected on the cytoplasm in gastric cancer cells as other report suggested [35], we were surprised to observe that CD24 was localized to the cell membrane, where it was partially co-localized and interacted with EGFR. This suggests that CD24 might have wider roles in cellular processes, such as modulating EGFR membranous stability. EGFR signaling pathway activation is primarily mediated by activated EGFR located in the plasma membrane [36]; then, several different downstream signaling pathways are activated. Interestingly, EGFR was found to be co-localized with another membrane glycoprotein CD44 in lipid rafts, and such co-localization could trigger downstream ERK and Ca^2+^/CaMKII activation, which were essential for TGF-β-stimulated fibroblast to myofibroblast differentiation [37]. The PI3 K/Akt and ERK pathways are important among them and have been implicated in cancer cell growth and migration [38]. Interestingly, recent studies showed that the downregulation of membranous E-cadherin is mediated by Akt or ERK activation [39, 40]. In this report, SGC-7901 gastric cancer cells depleting CD24 showed significant reductions in phosphorylation of ERK and Akt proteins as well as increases in E-cadherin expression levels. Moreover, E-cadherin was re-localized at cell-to-cell contacts and cell motility was reduced in those cells. Overall, the fact that CD24 interacted with EGFR and was crucial for activation of EGFR downstream effectors and cell migration, allowing us to conclude that regulation of EGFR stabilized expression can thus be a novel mechanism for CD24-induced gastric cancer progression.

Endocytosis of EGFR occurs in either a ligand-dependent or a ligand-independent way. Under normal conditions (serum-containing medium), EGFR can be constitutively internalized in the absence of EGF. After internalization, receptors are either recycled back to the cell surface, or sorted into intraluminal vesicles and subsequently delivered to lysosome for degradation to terminate EGFR activation [41, 42]. In breast cancer cells, CD24 is suggested to increase HER2 expression by transcriptional machineries via NF-κB signaling [21]. Here, we identified that CD24 maintains EGFR expression via reduction of EGFR internalization and degradation, but not by inhibition of its production. Another phenomenon we noticed here was that, in serum-starved conditions, CD24-knockdown cells had lower EGFR expression compared to control cells, and such difference was apparent before and after the gastric cancer cells were stimulated with EGF. In accordance with this, the Akt and ERK pathways are less activated by EGF in those cells. This result suggests that CD24 may regulate the basal level of EGFR. Notably, plasmatic EGFR staining was more visible and concentrated in CD24 knockdown cells in spite of EGF treatment. This lends support to the idea that CD24 may prevent EGFR internalization and degradation in the cytoplasm by an EGF-dependent or EGF-independent way.

How CD24 maintains EGFR expression on the cell surface remains unknown. On the membrane, EGFR and CD24 share interactions with several proteins including the non-receptor tyrosine kinase Src [15, 43–45]. CD24 is well known as the regulator of Src activity and Src-induced STAT3 and FAK activity [15, 16]. Recent work has also highlighted a co-localization between Src with EGFR, particularly within lipid rafts of cancer cells [46], Moreover, it was reported that Src could phosphorylate EGFR [47, 48], and mediate EGFR signaling in various cancer cell lines [46, 49]. However, in our study for SGC-7901 cells, loss of Src activation is unlikely to alter the kinetics of EGFR expression greatly. Therefore, additional proteins or molecules that play roles in the association between CD24 and EGFR remain to be identified. Given that the Rho GTPase subpopulation is thought to regulate EGFR endocytosis and trafficking [50], it appeared that Rho GTPases may be involved in the regulating process.

Three most common members of the Rho family are Rac1, Cdc42 and RhoA. All of them were recorded to participate in cellular endocytotic and recycling pathways. For example, activated Rac1 binds to F-actin cross-linking protein IQGAP1, and then inhibits the endocytosis of E-cadherin [51]. Cdc42 participates in the transport of glycoprotein of vesicular stomatitis virus (VSV-G) from the ER into the Golgi apparatus [52]. RhoA reportedly mainly participates in endocytic transport regulation [53, 54]. In some cell types, RhoA/ROCK is a downstream target of EGFR activation, which then turns off the activated EGFR pathway via a negative feedback system [55]. However, when we analyzed the role of RhoA protein on EGFR stability in gastric cancer cells, we observed that the inhibition of RhoA impaired EGFR expression and active RhoA markedly increased EGFR expression. Importantly, CD24-knockdown cells had lower RhoA activation compared to control cells. In accordance with this, a mathematical model has shown that over-expressing RhoA as well as its downstream effector ROCK could prolong ERK activation partly by reducing EGFR endocytosis [56]. Similarly, RhoA is required for delayed EGFR degradation in Vav2-overexpressing HeLa cells stimulated with EGF [57]. Mierke CT et al. have also reported that lung cancer cell invasiveness was reduced after pre-treatment with ROCK inhibitor Y27632 in CD24^high^ cells, but not in CD24^neg^ cells [58]. Indeed, RhoA and ROCK have been shown to be negatively associated with EGFR endocytosis. Early studies indicated that the active form of ROCK can inhibit the recruitment of endophilin A1 to the EGFR-c-Cbl-CIN85 complex, leading to the reduction of EGFR endocytosis in PC-12D cells [30]. Hence, the observation here explained, at least in part, the requirement of RhoA activation for CD24-mediated stable EGFR expression.

Together, these data point to the ability of CD24 to regulate EGFR signaling by preventing EGFR internalization and degradation through RhoA. Given that CD24 is a major GPI-anchored and lipid rafts-associated protein, it is possible that it may recruit and restrict lipid raft-localization of EGFR on the membrane. Hence, in CD24-positive cancer cells, EGFR keeps its association with lipid rafts on the membrane that is necessary for its signaling transduction and reducing its internalization and degradation. These findings are of potential pathophysiological importance for understanding the integration of CD24-related signaling and shed light on anti-CD24 therapy for gastric cancer.

## Additional files

**Additional file 1: Figure S1.** Effect of CD24 overexpression on EGFR level in BGC-823 cells. BGC-823 cells were transfected with empty vector or CD24 plasmids, and the total cellular proteins were extracted and analyzed for expression of EGFR by Western blotting assays.

**Additional file 2: Figure S2.** CD24 forms complexes with EGFR. (A&B) Co-immunoprecipitation of CD24 by EGFR was determined. SGC-7901 cells (A) or BGC-823 cells (B) were immunoprecipitated with anti-EGFR antibody, followed by Western blotting assays for CD24. The second top panel shows immunoprecipitated CD24. The third top panel shows GAPDH bands in samples of input and immunoprecipitation. After pulldown, the supernatant was also subjected to Western blotting assays to detect the expression of GAPDH. (C) Representative micrographs of BGC-823 cells stained for CD24 (red) and EGFR expression (green) by immunofluorescence staining. The arrow shows that co-location of CD24 and EGFR. Scale bar, 10 μm. n = 3 for all experiments.

**Additional file 3: Figure S3.** Effect of EGFR inhibitor erlotinib on P-Akt and P-ERK levels in CD24 overexpressed GES-1 cells. GES-1 cells transfected with empty vector or CD24 plasmids were incubated with 1 μM erlotinib, or the cells in serum-free media overnight, then the cells were treated with EGF (20 ng/mL) for 20 min after erlotinib incubation. Proteins extracted from the lysates were subjected to Western blotting assays to detect the expression of P-Akt and P-ERK.

**Additional file 4: Figure S4.** Effect of CD24 silencing on EGF-induced phosphorylation of ERK and Akt. SGC-7901 cells transfected with control siRNA or siCD24 were cultured in serum-free media overnight and incubated with EGF 20 ng/mL for indicated times, and then the proteins extracted from the lysates were subjected to Western blotting assays to detect the expression of proteins as indicated.
