# Supplementary figures and images for: CD24 associates with EGFR and supports EGF/EGFR signaling via RhoA in gastric cancer cells
Source: J Transl Med. 2016 Feb 1;14:32. doi: 10.1186/s12967-016-0787-y (PMC5439121; doi:10.1186/s12967-016-0787-y)

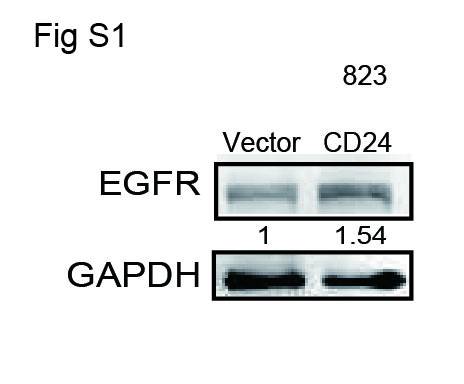

Supplement: Supplementary file 1 — Additional file 1: Figure S1. Effect of CD24 overexpression on EGFR level in BGC-823 cells. BGC-823 cells were transfected with empty vector or CD24 plasmids, and the total cellular proteins were extracted and analyzed for expression of EGFR by Western blotting assays. [file 12967_2016_787_MOESM1_ESM.jpg]

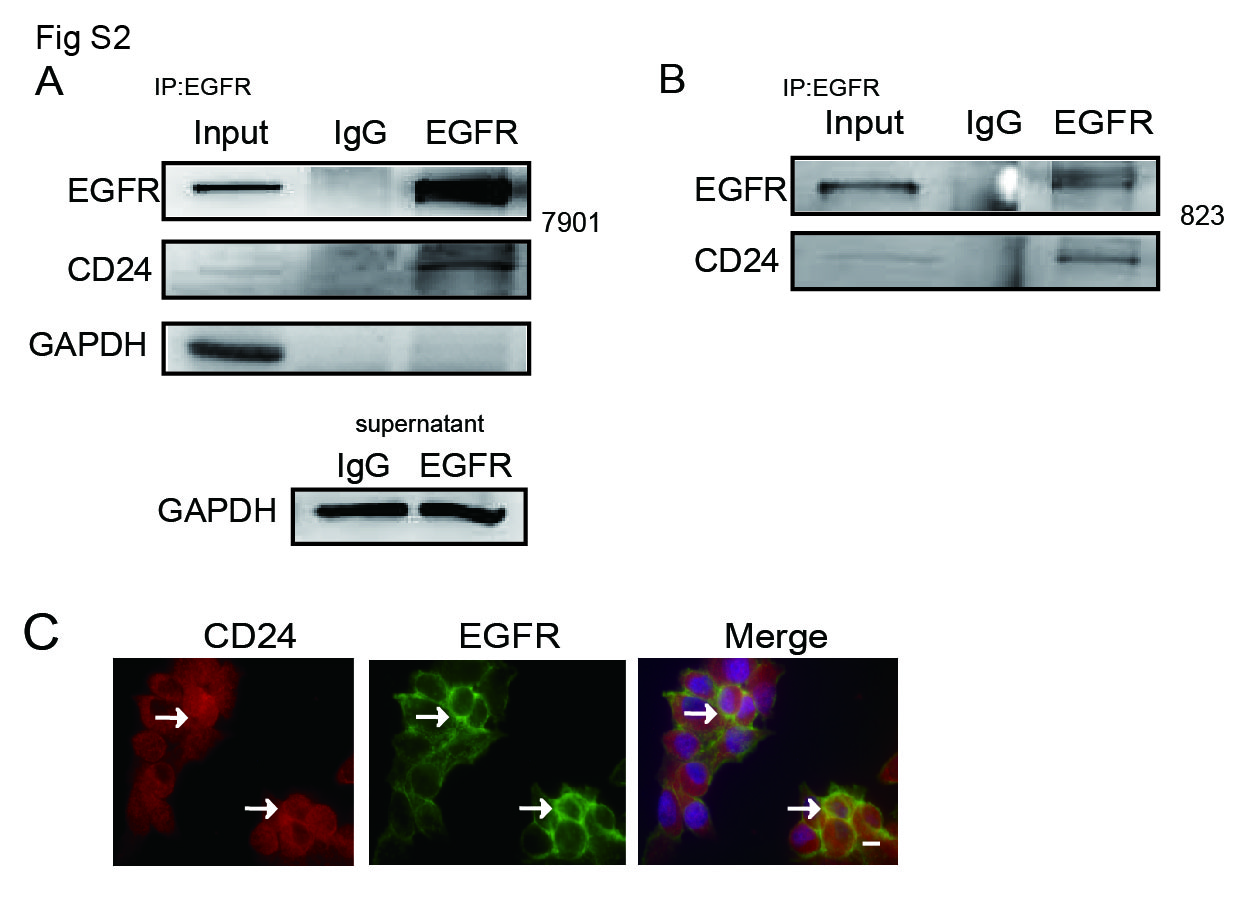

Supplement: Supplementary file 2 — Additional file 2: Figure S2. CD24 forms complexes with EGFR. (A&B) Co-immunoprecipitation of CD24 by EGFR was determined. SGC-7901 cells (A) or BGC-823 cells (B) were immunoprecipitated with anti-EGFR antibody, followed by Western blotting assays for CD24. The second top panel shows immunoprecipitated CD24. The third top panel shows GAPDH bands in samples of input and immunoprecipitation. After pulldown, the supernatant was also subjected to Western blotting assays to detect the expression of GAPDH. (C) Representative micrographs of BGC-823 cells stained for CD24 (red) and EGFR expression (green) by immunofluorescence staining. The arrow shows that co-location of CD24 and EGFR. Scale bar, 10 μm. n = 3 for all experiments. [file 12967_2016_787_MOESM2_ESM.jpg]

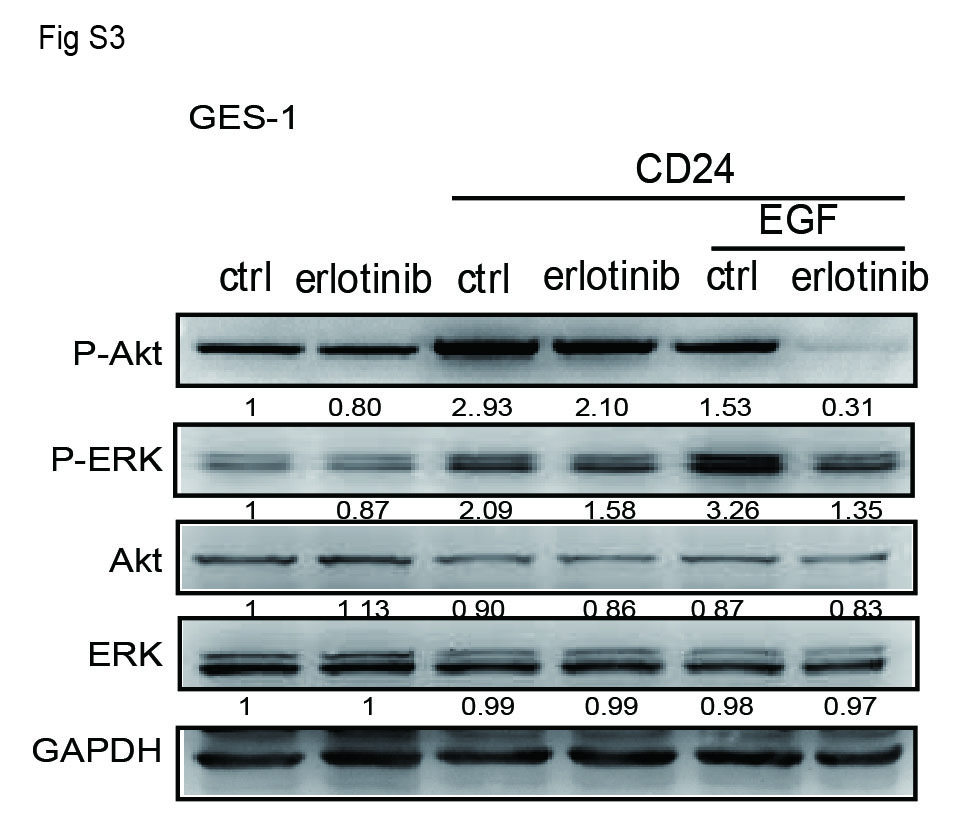

Supplement: Supplementary file 3 — Additional file 3: Figure S3. Effect of EGFR inhibitor erlotinib on P-Akt and P-ERK levels in CD24 overexpressed GES-1 cells. GES-1 cells transfected with empty vector or CD24 plasmids were incubated with 1 μM erlotinib, or the cells in serum-free media overnight, then the cells were treated with EGF (20 ng/mL) for 20 min after erlotinib incubation. Proteins extracted from the lysates were subjected to Western blotting assays to detect the expression of P-Akt and P-ERK. [file 12967_2016_787_MOESM3_ESM.jpg]

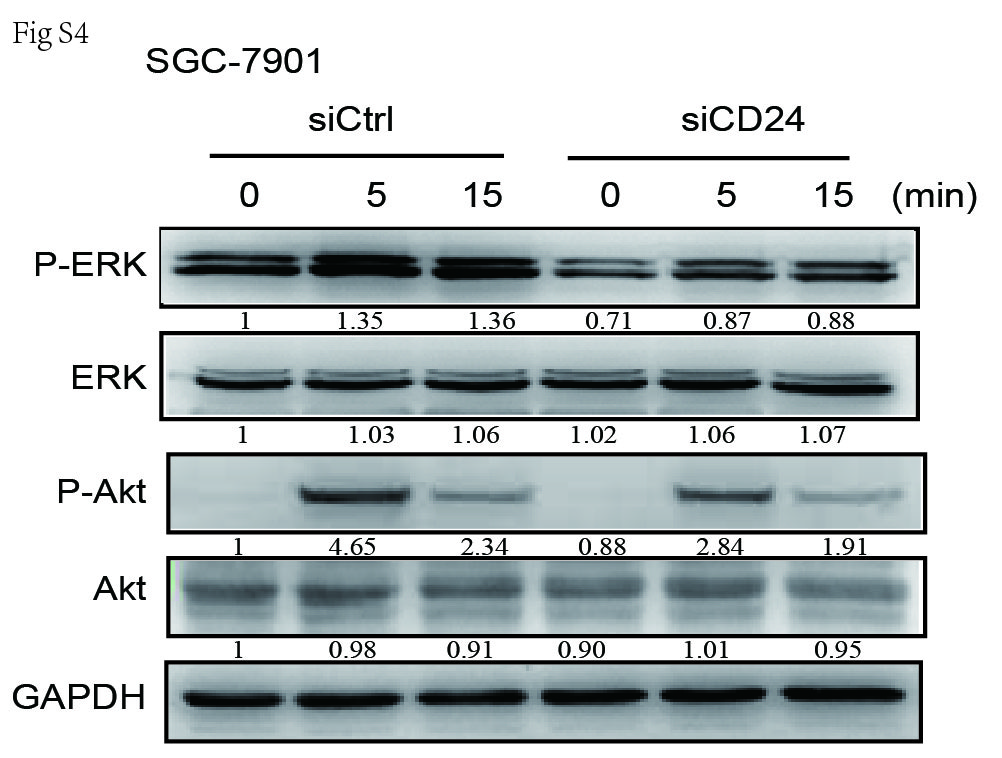

Supplement: Supplementary file 4 — Additional file 4: Figure S4. Effect of CD24 silencing on EGF-induced phosphorylation of ERK and Akt. SGC-7901 cells transfected with control siRNA or siCD24 were cultured in serum-free media overnight and incubated with EGF 20 ng/mL for indicated times, and then the proteins extracted from the lysates were subjected to Western blotting assays to detect the expression of proteins as indicated. [file 12967_2016_787_MOESM4_ESM.jpg]
